# Time-resolved characteristics of deuteron-beam generated by plasma focus discharge

**DOI:** 10.1371/journal.pone.0188009

**Published:** 2018-01-08

**Authors:** Lian-Kuang Lim, Seong-Ling Yap, D. A. Bradley

**Affiliations:** 1 Plasma Technology Research Centre, Physics Department, Faculty of Science, University of Malaya, Kuala Lumpur, Malaysia; 2 Institute for Healthcare Development, Sunway University, Selangor, Malaysia; 3 Department of Physics, Faculty of Engineering and Physical Sciences, University of Surrey, Guildford, United Kingdom; Ludwig-Maximilians-Universitat Munchen, GERMANY

## Abstract

The plasma focus device discussed herein is a Z-pinch pulsed-plasma arrangement. In this, the plasma is heated and compressed into a cylindrical column, producing a typical density of > 10^25^ particles/m^3^ and a temperature of (1–3) × 10^7 o^C. The plasma focus has been widely investigated as a radiation source, including as ion-beams, electron-beams and as a source of x-ray and neutron production, providing considerable scope for use in a variety of technological situations. Thus said, the nature of the radiation emission depends on the dynamics of the plasma pinch. In this study of the characteristics of deuteron-beam emission, in terms of energy, fluence and angular distribution were analyzed. The 2.7 kJ plasma focus discharge has been made to operate at a pressure of less than 1 mbar rather than at its more conventional operating pressure of a few mbar. Faraday cup were used to determine deuteron-beam energy and deuteron-beam fluence per shot while CR-39 solid-state nuclear track detectors were employed in studying the angular distribution of deuteron emission. Beam energy and deuteron-beam fluence per shot have been found to be pressure dependent. The largest value of average deuteron energy measured for present conditions was found to be (52 ± 7) keV, while the deuteron-beam fluence per shot was of the order of 10^15^ ions/m^2^ when operated at a pressure of 0.2 mbar. The deuteron-beam emission is in the forward direction and is observed to be highly anisotropic.

## Introduction

The plasma focus device is a coaxial plasma accelerator in which the electrical energy that is stored in the capacitor bank is rapidly converted into the formation of a hot plasma at the end of the coaxially arranged electrodes. Upon high voltage breakdown, a plasma current sheath is formed at the breech of the electrodes assembly. As a result of the Lorentz force this is accelerated along the electrodes until at the open end formation occurs of a short-lived pinched-plasma column that is of high density, at > 10^25^ particles/m^3^, and very high temperature ~ (1–3) × 10^7 o^C [1]. The transient hot and dense plasma is of particular interest due to the multitude of physical phenomena, typically magnetohydrodynamic instabilities, producing an intense burst of energetic and multi-radiation emissions, including x-rays, ions and electrons [2–4]. Previous University of Malaya research on this plasma tended to concentrate on the plasma focus as a neutron source [5], also in studies of the neutron production mechanism [6–10], related in particular to the investigation of the thermonuclear origin. Most results pointed to the conclusion that the plasma focus is a low feasibility source for thermonuclear fusion research, also with no great part seen in working towards an effective fusion energy solution. Thus said, at high current operation of more than 10 MA theoretical work has reported the possibility of energy break-even [11]. As an alternative scheme for clean energy, encouraging results making use intense energetic ion-beam have also been reported [12].

Substantial efforts have been undertaken in investigating ion-beam emission [13–15], with the detailed mechanisms responsible for the emission remaining to be understood. This is due to difficulties in the design of diagnostic techniques and detectors having sufficient temporal and spatial resolution to record the transient nature and wide energy range of the emitted ion-beam. Existing diagnostics techniques that are often used to measure the ion-beam characteristics are the Thomson parabola Spectrometer [16], Magnetic spectrometer [17], nuclear activation method [18], Faraday cup [19, 20], biased ion collectors [21], solid state nuclear track detectors (SSNTDs) [22] and activation yield-ratio technique [23].

In their time of flight (ToF) measurements making use of a Faraday cup, Bostick et al. [24] showed deuterons of energy 1–9 MeV to have lower deuteron-beam fluence, at ~10^12^ (MeV.sr)^-1^, compared to deuterons of lower energy (300–500 keV), with fluence of ~10^14^ (MeV.sr)^-1^. Sadowski et al. [25] compared the ion-beam characteristics obtained from different plasma focus devices operating with an input energy of between 5–50 kJ. It was concluded that deuterons of > 100 keV are emitted from the anode axis within the range of solid-angles 60°–80° and that the intensity of the ion-beam to be highly dependent on the electrode geometry, input energy, operating pressure and types of working gas. At one large plasma focus facility operated at 1 MJ, the ion-beam emission was also found to be mostly directed towards the anode end-on direction, with energies >100 keV and fluxes of 10^11^ ions/m^2^ [26].

In regard to ion-beam emission, Mozer et al. [27] reported more than one acceleration process to be taking place. Szydlowski et al. (24) and Zakaullah et al. [28] investigated the angular distribution of deuterons using solid state nuclear track detectors (SSNTDs), revealing strong anisotropy. Similar observation was also reported by Mohanthy et al. [29] using a multi Faraday cup assembly in study of a nitrogen filled plasma focus device. Sadowska et al. [30] explained the anisotropy of the deuteron angular distribution to be due to the stochastic character of formation of ion micro-sources within the plasma column.

Experiments apart, Lee et al. [31] developed and incorporated a fluence equation into their model of the ion-beam, computing beam properties through a curve-fitting technique. An empirical method was employed, results being extracted by fitting the measured current signal to that computed, the model being developed to allow use of various gases, intending to provide guidelines in material applications.

Progress in such research has stimulated ion-beam emission studies in areas of material science, the ion-beam source motivating research applications in for instance surface modification [32, 33], ion implantation [34, 35], thin-film deposition [36, 37], semiconductor doping [38], synthesis of nanoparticles [39], amorphization of silicon [40], Nanostructuring of FePt thin films [41] and formation of nanoparticle [42]. While many ion-beam measurement and diagnostic techniques have been developed, most particularly in seeking to characterize ion-beam emission, challenges continue to remain in clarifying the various emission complexities. With paucity in comparison of results from the different ion measurement techniques, present work implements a number of these techniques, comparing outcome and seeking further characterization of the deuteron-beam emission.

## Experimental setup

### Plasma focus device

**Fig 1:**
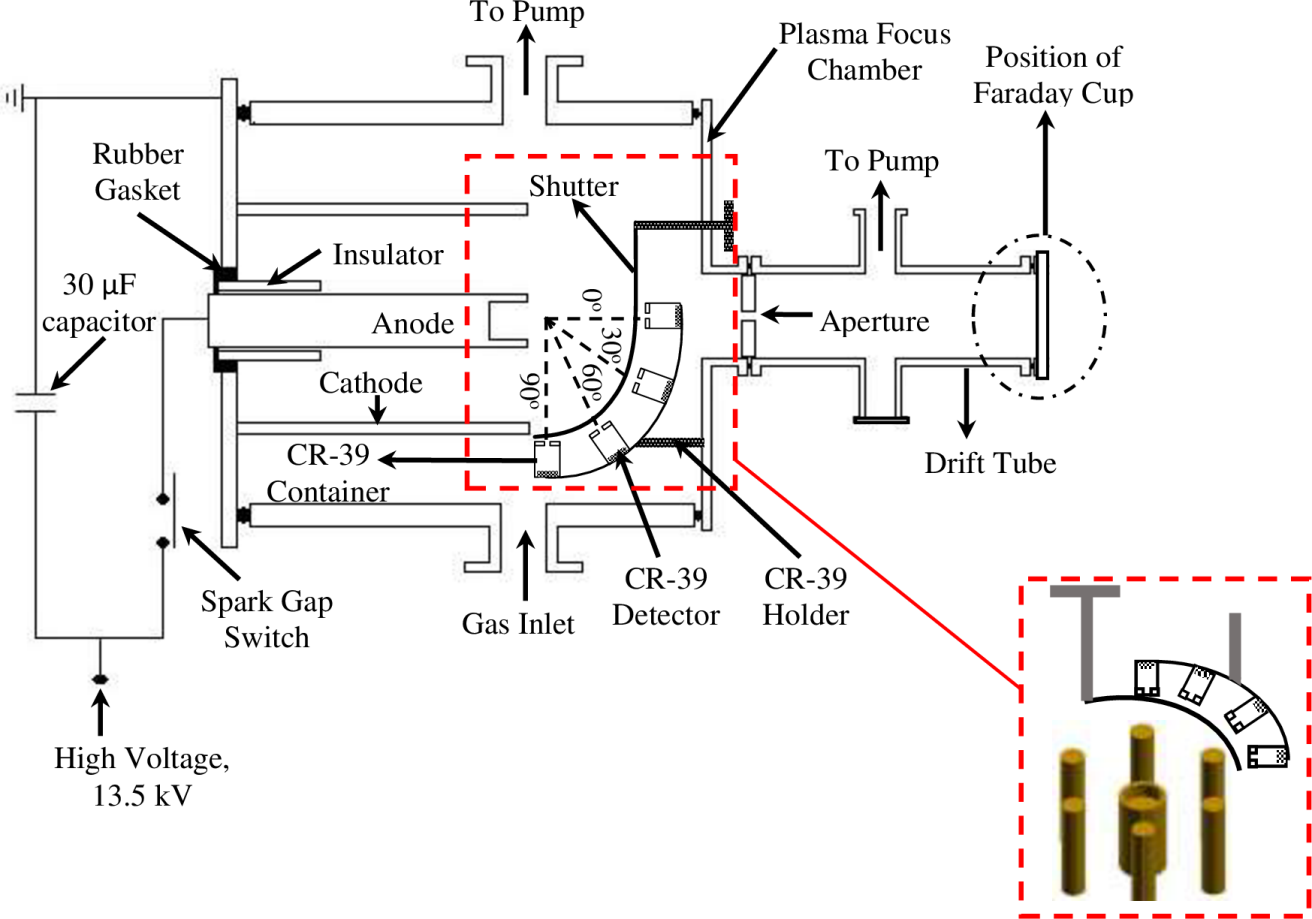
Schematic of the plasma focus device equipped with diagnostics of a Faraday cup, biased ion collectors and CR-39 SSNTDs.

In present work, a Mather type plasma focus device has been used, energized by a single low inductance 30 μF Maxwell capacitor delivering a maximum energy of 2.7 kJ when discharged at 13.5 kV. The plasma focus tube electrode assembly comprises six outer electrodes (the cathode) concentrically arranged at a radius of 3.2 cm from an inner electrode (the anode) immersed in a cylindrical chromed mild steel vacuum chamber of diameter 15 cm and height of 30 cm. Both electrodes were made of Cu tube, length 22 cm. The outer diameter of the anode is 19 mm with the open end engraved to a depth of 4 cm. Between the anode and cathode, the electrodes were separated by a hollow cylindrical Pyrex insulator sleeve of length 50 mm. With this configuration, the operating pressure was reduced to < 1 mbar to match the characteristic time of the electrical discharge, for the operation pressures of 0.1 mbar to 1.0 mbar in deuterium filling. For these low filling pressures, in order to enhance the initial breakdown of the current sheath at the surface of the insulator sleeve, a set of twelve injection plasma guns was installed surrounding the cathodes. The tangential arrangement of the plasma guns also helps to initiate an axisymmetric current sheath during the breakdown phase. Fig 1 provides a schematic of the plasma focus device, together with the diagnostics tools used.

## Diagnostic techniques

In the present work deuterium was used as the working gas. To examine the deuteron-beam emission versus operating pressure the plasma focus has been discharged at different deuterium filling pressures, from 0.1–1.0 mbar. At this low pressure regime, ion beam emission with high fluence and energy can be obtained due to the anomalous resistivity effect.

The values of discharge current and voltage were measured with a Rogoswki coil and a resistive voltage divider respectively. Time-resolved measurements of the deuteron-beam were carried out employing the Faraday cup.

The Faraday cup, a circular Cu disk charge collector housed inside a cylindrical brass cage, was mounted on a drift tube installed in the end-on direction. Separation from the anode tip of some 0.4 m provided for measurement of deuteron beam fluence, picking up the beam impinging the collector detector. The drift tube was connected to the main chamber via a 2 mm diameter aperture, the pressure inside the drift tube being kept at < 10^−5^ mbar to reduce scattering and charge neutralization of the deuteron-beam. A gate-valve was installed separating the plasma focus chamber and the drift tube. The valve is closed except during discharge to maintain the pressure difference between the plasma focus chamber and the drift tube.

Time integrated measurements of deuteron-beam emission were carried out with the use of CR-39 SSNTDs, the track detectors being chosen due to their high sensitivity to high-energy ions, low sensitivity towards electrons and electromagnetic radiation and a capability to detect single ions with energies up to a few MeV. The SSNTDs, of dimension (1 cm x 1 cm x 1 mm), were cut from an initial larger area of CR-39 medium and then located on a special quarter-circular support to provide for simultaneous investigation of the angular distribution of deuteron-beam at four orientations: 0° (end-on), 30°, 60° and 90° (side-on). All the CR-39 detectors, placed at the same radial distance of 5.7 cm from the anode tip, were housed inside a small cylindrical brass container. A pinhole at one end of the container allowed for entrance of the deuteron-beam. Prior to measurement of deuteron-beam emission angular distribution, use was made of an Al shutter formed from a strip 0.6 mm thick in order to provide effective shielding of the SSNTDs. Subsequent to exposure, the CR-39 nuclear track detectors were etched for 5 hours in 6.0 mol dm^-3^ NaOH solution maintained at 60° C.

## Results and discussions

**Fig 2:**
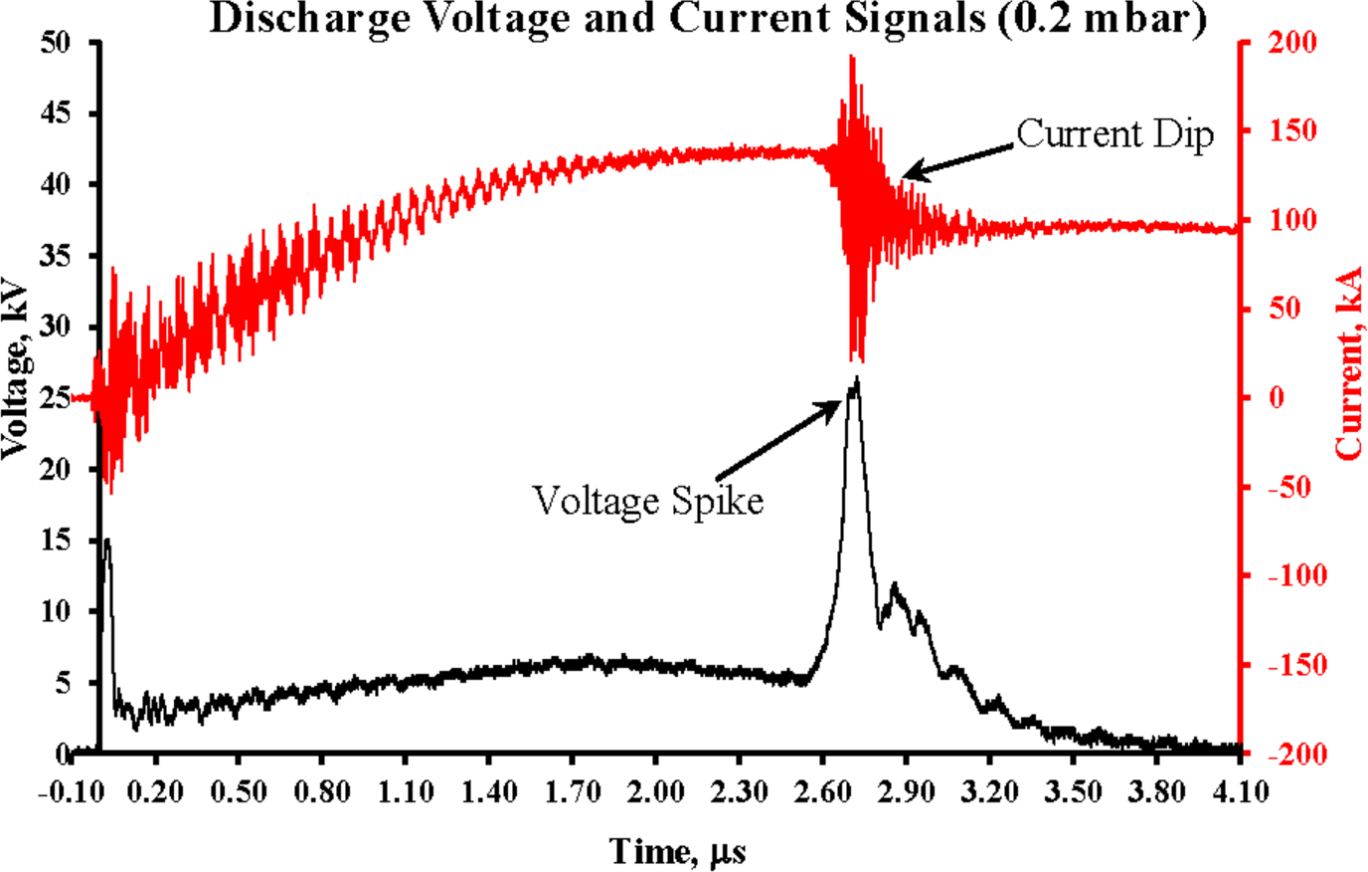
Discharge voltage and current signal at 0.2 mbar deuterium.

Results of measurements of the Faraday cup and SSNTDs (CR-39) have been cross-compared. Fig 2 shows a typical pair of discharge voltage and current signals, representing a tightly-pinched discharge. The pinching phenomenon, otherwise referred to as the plasma focus action, is one characterized by a sharp rise in the voltage (the voltage spike), simultaneous with a significant dip in the overall current signal. The plasma focus action has been observed to be maximal at low operating pressures, at 0.2 mbar, the focus action occurring near to the peak of maximum discharge current, associated with greatest transfer of energy to the pinched plasma. This condition, consistent with strong focusing discharge, is followed by intense deuteron beam emission.

## Measurements of deuteron beam

**Fig 3:**
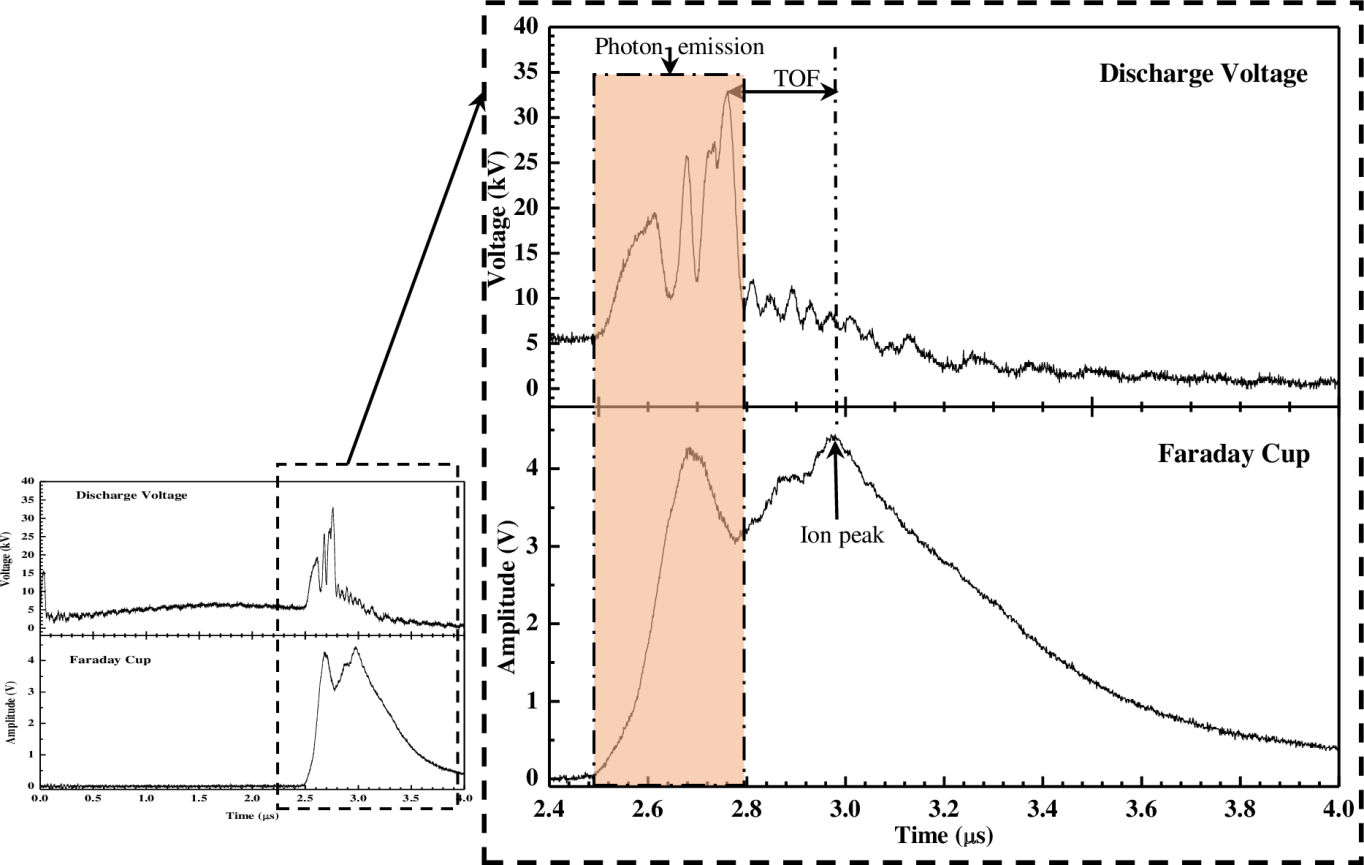
Typical discharge voltage and Faraday cup signal at 0.2 mbar deuterium.

Fig 3, obtained with the Faraday cup, shows measurement of deuteron-beam emission together with the discharge voltage signal, the voltage spike being used as indication of the initiation of the deuteron-beam.

The first peak in the Faraday cup signal coincided with that of the voltage spikes, attributable to strong photon emission pick-up during the pinch formation, the Faraday cup also being able to pick up photons produced by the photoelectric effect. The second peak is the deuteron-beam signal. The deuteron-beam velocity has been estimated through the use of the ToF technique, simply the ratio of the separation distance to ions flight time, from the emission region to the Faraday Cup. The ion flight time is determined by taking the time difference from the peak of voltage spike to the highest peak in the Faraday cup signal. From the Faraday cup signal obtained at 0.2 mbar, the average deuteron-beam energy over 5 shots was determined to be (52 ± 7) keV while the ToF deuteron-beam energy estimation for the particular shot was found to be about (38 ± 1)keV.

**Fig 4:**
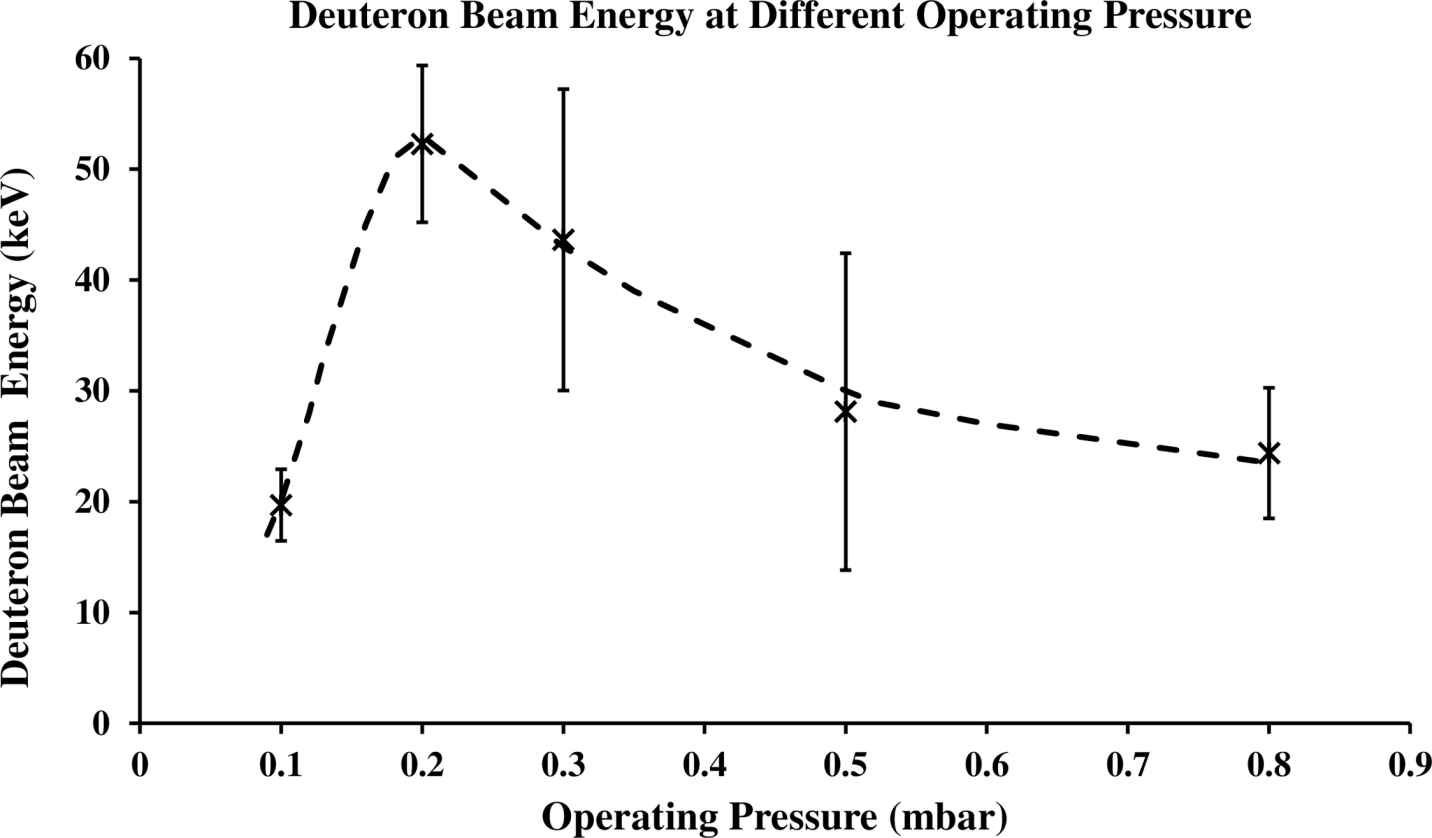
Average deuteron-beam energies at different operating pressures using Faraday cup.

The average deuteron-beam energies over 5 shots, determined for other operating pressures as investigated herein, are plotted in Fig 4, varying with operating pressure and peaking at 0.2 mbar, a behavior correlating with the focusing action of the discharge. At a pressure of 0.8 mbar and beyond, weak focusing discharge has been obtained. In reducing the operating pressure from 0.8 mbar, the amplitude of the voltage spike and current dip has been observed to increase, in line with stronger focusing discharge. Focusing discharge at the greatest voltage spike amplitude, also associated with the noted significant dip in current signal, is consistently observed at 0.2 mbar. The strong focusing discharge is suggestive of induction of very high local electric field strength, accelerating the deuteron-beam to high energy. The focusing action was weaker for pressures < 0.2 mbar, occurring before the discharge current increased to a maximum.

**Fig 5:**
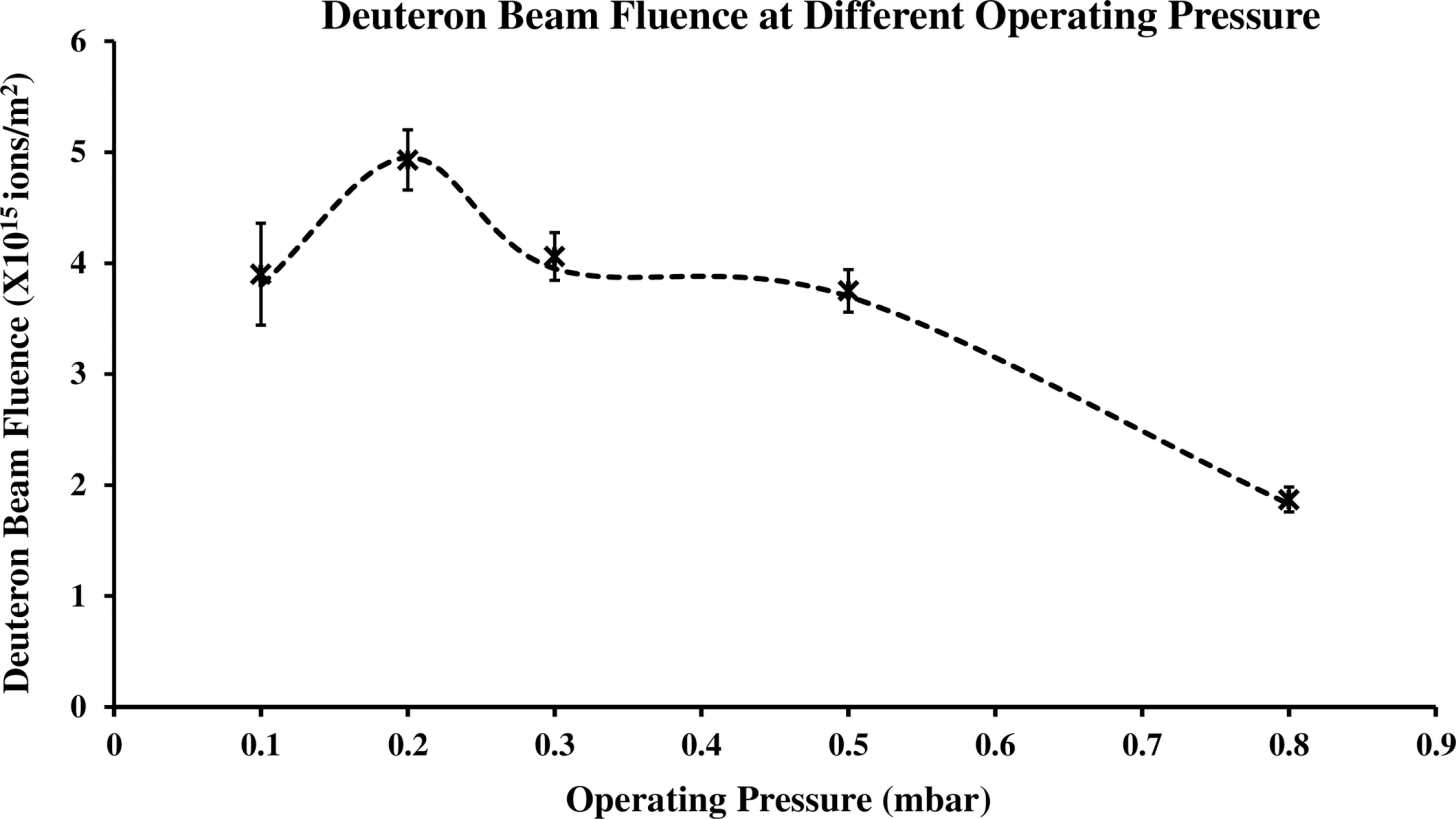
Average deuteron-beam fluence per shot at different operating pressure.

Based on Faraday cup measurements, determination has been made of the deuteron-beam fluence (number of deuterons per unit area) per shot along the end-on direction. Despite the de-merits of low signal to noise ratio and the effect of secondary electron emission, the Faraday cup has the ability to collect all of the incoming charged particles directed to its collector. The total deuterons collected per unit detector area are presented in Fig 5, obtained by integrating the Faraday cup signal with respect to time and excluding the part of signal contributed by photon emission. Each data point on the graph was the average obtained over 5 shots. Clearly shown is that the deuteron-beam fluence is also dependent on the operating pressures. An average deuteron-beam fluence of the order of 10^15^ ions/m^2^ was estimated, with the greatest fluence obtained at 0.2 mbar.

Similar trends with operating pressure are observed in the deuteron-beam energy and deuteron-beam fluence per shot. From the overall results, 0.2 mbar is found to be the optimal operating pressure, favourable for deuteron-beam emission in terms of beam energy and deuteron-beam fluence. The focusing discharge was observed to be highly dependent on the operating pressure, indicating the possibility to tune the operating pressure to a required deuteron-beam characteristic.

## Particle measurement with CR-39 SSNTDs

**Fig 6:**
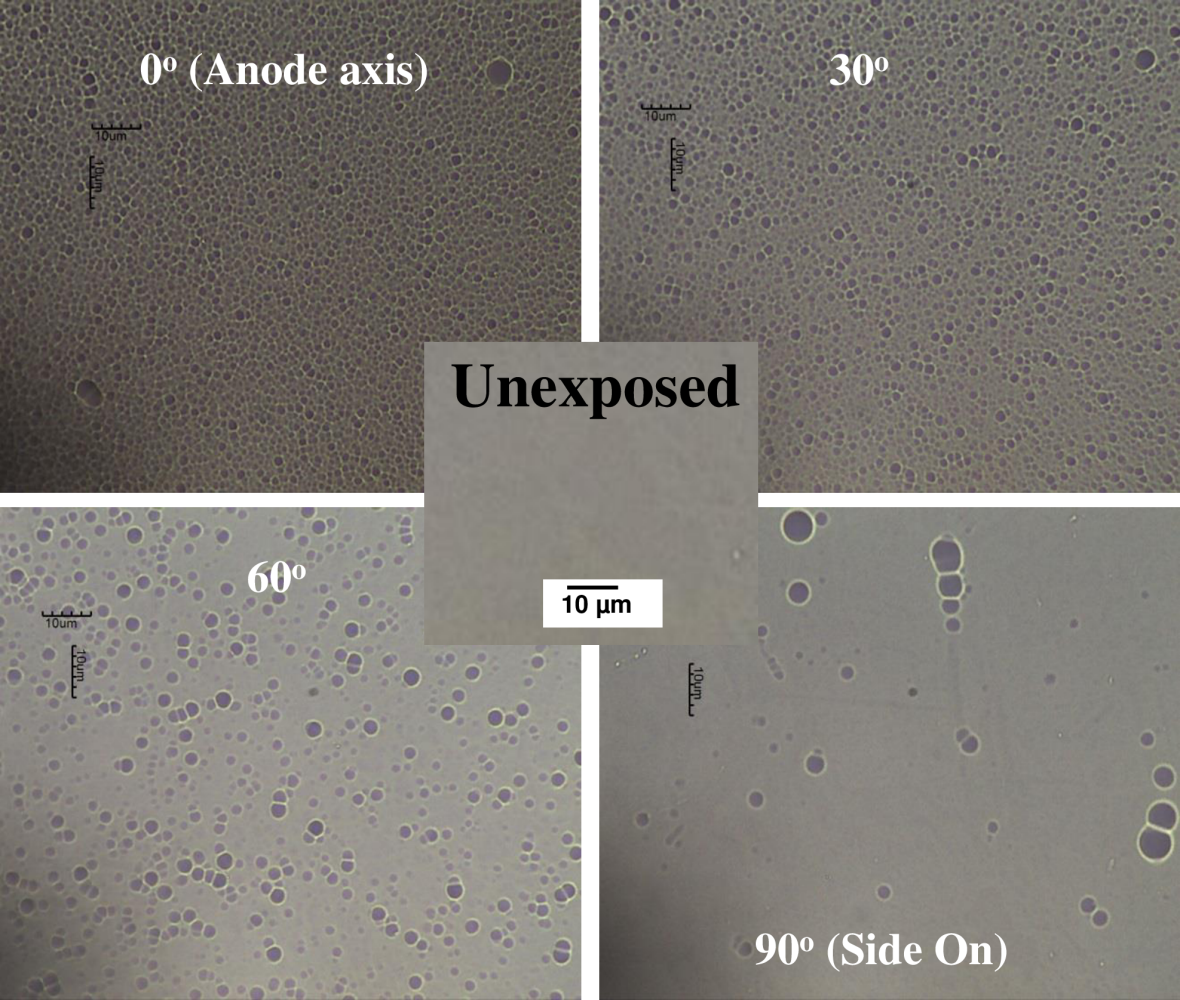
Optical microscope images (1000X) of CR-39 SSNTDS exposed at various angular positions, at 0.2 mbar deuterium.

Fig 6, obtained through simultaneous exposures of CR-39 SSNTDs located at various angular orientations with respect to the forward direction, shows strong variation in angular distribution of deuteron-beam emission, the total number of deuteron tracks registered at each angular position showing distinct anisotropy. Forward emission is favoured as expected, with the density of deuteron tracks markedly decreasing with increase in angular position. The deuteron track density registering in the end-on direction is also seen to approach towards saturation (with numerous examples of etch pits overlapping), one indication from this being that the density of deuterons exceeds 10^11^ ions/m^2^. At the angular position of 30° there are lesser numbers of registered deuteron tracks, albeit still at significant deuteron track density and occurrence of etch pit overlap. At the angular position of 60° strong reduction in etch pit density is notable. However also observed together is an increase in opening size of the deuteron track etch pits, indicative of greater, more superficial, energy loss (linear-energy transfer, LET,—*dE*/*dx*) at these larger angles of incidence onto the detector surface, becoming even more pronounced for deuteron tracks registered at 90°, with much fewer deuterons also being registered for this orientation of detector. However it is to be appreciated here that the SSNTDs are incapable of detection beyond some critical incident angle (the value of which depends on LET as well as etching conditions). This occurs at an angle at which the track etching rate (the rate of etching along the track) is matched by the surface bulk etching rate (the rate at which the surface is etched away) so that the etch pit never become visible. This adds to the apparent reduction in track density with angle such that the use of SSNTDs provides only for qualitative evaluation of the angular emission dependency rather than an absolute measure. For a fuller account of this situation see [43].

Measurement of the deuteron tracks etch pit size at each angular position has been recorded in terms of the average size measured for a minimum of 100 deuteron tracks at different exposed areas on the detector, except at the angular position of 90°. Average etch pit openings (the surface diameter of the deuteron track craters) at 0°, 30°, 60° and 90° were measured to be 1.7 ± 0.3 μm, 1.9 ± 0.3 μm, 2.3 ± 0.4 μm and 3.7 ± 1.0 μm, respectively. Due to the saturated deuteron track density along the end-on direction, the anisotropy factor of deuteron-beam emission was not able to be determined.

Overall results show the CR-39 SSNTDs to be convenient for qualitative evaluation of deuteron angular distribution. Among the de-merits in use of a passive detector system such as CR-39 includes extended processing times to provide for final assessment (in the case of the SSNTDs, including etching time and cleaning steps before analysis can be made of the deuteron tracks under an optical microscope). SSNTDs also suffer from saturation when deuteron track density becomes too great to avoid substantial deuteron tracks overlap, as in a detector positioned in the end-on direction, as well as there being a critical angle above which tracks will no longer be formed. Nevertheless, CR-39 nuclear track detectors are a relatively cheap ion beam diagnostic system, also simple in application when compared to the Faraday cup systems.

## Conclusion

Experimental studies of deuteron beam emission from a plasma focus operated at low pressure regime has been carried out with different diagnostic techniques including Faraday cup and CR-39 nuclear track detectors. The results analyzed give the characteristic of deuteron beam in terms of beam energy, beam fluence per shot as well as angular distribution of deuteron beam emission.

In the low operation pressure regime of below 1 mbar deuteron-beam fluence of the order of 10^15^ ions/m^2^ has been determined from the Faraday cup measurement. The best operating pressure in that regime gives maximum fluence at 0.2 mbar. Angular distribution measurement using CR-39 shown the intense deuteron beam emission was observed within solid angle of 60°. The maximum average deuteron beam energy of 52 ± 7 keV was determined and was found to be strongly dependent on the operating pressure. The deuteron beam can be potentially applied in the studies of beam-target reaction. Beam target fusion production of neutron with deuteron with the average energy of 20–60 keV has been reported [44].
